# Novel findings associated with *MTM1* suggest a higher number of female symptomatic carriers

**DOI:** 10.1016/j.nmd.2016.02.004

**Published:** 2016

**Authors:** Marco Savarese, Olimpia Musumeci, Teresa Giugliano, Anna Rubegni, Chiara Fiorillo, Fabiana Fattori, Annalaura Torella, Roberta Battini, Carmelo Rodolico, Aniello Pugliese, Giulio Piluso, Lorenzo Maggi, Adele D'Amico, Claudio Bruno, Enrico Bertini, Filippo Maria Santorelli, Marina Mora, Antonio Toscano, Carlo Minetti, Vincenzo Nigro

**Affiliations:** a Dipartimento di Biochimica, Biofisica e Patologia Generale, Seconda Università di Napoli, Napoli, Italy; b Telethon Institute of Genetics and Medicine, Pozzuoli, Italy; c Dipartimento di Neuroscienze, Università degli Studi di Messina, Messina, Italy; d IRCCS Fondazione Stella Maris, Pisa, Italy; e IRCCS Ospedale Pediatrico Bambino Gesù, Roma, Italy; f A.O.R.N.A. Cardarelli, Napoli, Italy; g Dipartimento di Neuroscienze, Istituto Besta, Milano, Italy; h Center of Myology and Neurodegenerative Disorders, Istituto Giannina Gaslini, Genova, Italy

**Keywords:** X-linked myotubular myopathy, *MTM1* gene, Abnormal genital development, Next-generation sequencing, CGH array

## Abstract

Mutations in the *MTM1* gene cause X-linked myotubular myopathy (XLMTM), characterized by neonatal hypotonia and respiratory failure, and are responsible for a premature mortality in affected males. Female carriers are usually asymptomatic but they may present with muscular weakness because of a hypothesized skewed pattern of X-chromosome inactivation.

By combining next generation sequencing (NGS) and CGH array approaches, we have investigated the role of *MTM1* variants in a large cohort of undiagnosed patients with a wide spectrum of myopathies. Seven novel XLMTM patients have been identified, including two girls with an unremarkable family history for myotubular myopathy.

Moreover, we have detected and finely mapped a large deletion causing a myotubular myopathy with abnormal genital development.

Our data confirm that the severe neonatal onset of the disease in male infants is sufficient to address the direct molecular testing toward the *MTM1* gene and, above all, suggest that the number of undiagnosed symptomatic female carriers is probably underestimated.

## Highlights

• 504 myopathic patients have been screened for *MTM1* variants by NGS and CGH array approaches.
• Seven novel XLMTM patients and the fifth case of a large Xq28 deletion have been identified.
• The identification of two sporadic manifesting female carriers suggests that their number may be underestimated.
• Large NGS panels, including the *MTM1* gene, are useful tools to identify sporadic female XLMTM patients.
• The identification of *MTM1* variants, also as incidental findings, complicates genetic counseling.

## 1 Introduction

Centronuclear myopathies (CNMs) are congenital myopathies characterized by the presence of nuclei in the central part of the muscle fibers [1]. Four genetically different types have been described so far: an autosomal dominant form caused by mutations in the *DNM2* gene [2]; an autosomal dominant or recessive form related to mutations in the *BIN1* gene [3], [4]; an autosomal dominant or recessive form caused by mutations in the *RYR1* gene [5]; and an X-linked myotubular myopathy (XLMTM) due to mutations in the *MTM1* gene [6].

The *MTM1* gene comprises 15 exons and codes for myotubularin, a phosphatase targeting specifically PtdIns3P and PtdInsP2, two phosphoinositides (PIs) involved in the endosomal–lysosomal pathway [7], [8]. Myotubularin is essential for muscle cell differentiation and regulates the mitochondrial morphology in muscular fibers by a direct interaction with desmin [9].

In 1996, mutations in the *MTM1* gene were identified as causative of the XLMTM condition, characterized by a variable, but usually severe, phenotype [6]. Hypotonia at birth, muscle weakness and respiratory failure causing a neonatal mortality occur in the most severe cases [10]. Its prevalence is nearly 1/50,000 males and female carriers are usually asymptomatic [11]. However, a number of carriers, manifesting a milder phenotype probably due to a skewed X inactivation, have been described. Considering the broad range of phenotypes caused by *MTM1* mutations, the presence of necklace fibers at muscle biopsy is a hallmark of this specific disease as well as of a DNM2 related myopathy [12], [13], [14].

As evidenced in recent mutation screenings of *MTM1*, small variants, including missense, nonsense and splice site single base changes, represent the largest proportion (93%) of causative mutations in this gene [11], [15]. In particular, some missense mutations are correlated to a milder phenotype and most nonsense variants or rare large deletions cause a severe condition.

Here we describe seven novel patients with causative point mutations in the *MTM1* gene and a family with a large deletion on the X chromosome, detected by performing a next generation sequencing (NGS) approach [16] and a customized CGH array analysis [17] in a large cohort of undiagnosed patients with a wide spectrum of myopathies.

## 2 Materials and methods

### 2.1 Sample collection

For the NGS screening, 504 DNA samples from patients (59.6% males) with a wide spectrum of myopathies, including a congenital myopathy (32.5%), a limb-girdle muscular dystrophy (LGMD - 51.3%) or other clinical conditions (16.2%, comprising distal myopathy, isolated hyperckemia and metabolic myopathy) were collected. A written informed consent was signed by patients, according to the guidelines of Telethon Italy and as approved by the Ethics Committee of the “Seconda Università degli Studi di Napoli”, Naples, Italy. More than 90% of samples collected had previously been tested unsuccessfully, according to the observed phenotype.

In 105 patients without any significant variant detected by NGS, a CNV analysis by a custom CGH array was also carried out.

### 2.2 Molecular analysis

Genomic DNA was extracted from the peripheral blood by phenol/chloroform extraction.

For the NGS screening, the samples were enriched using the MotorPlex assay, as previously described [16]. In all the samples analyzed, all the exons of the *MTM1* gene and the 10 flanking bases were sequenced at a coverage >20×.

The raw data obtained were analyzed using an in-house pipeline described elsewhere [16], [18].

*MTM1* mutated exons were amplified by PCR using M13-tailed primers. M13 primers were used to perform Sanger sequencing using an ABI PRISM 3130 XL automatic DNA Sequencer Genetic Analyzer (Applied Biosystems, Foster City, CA, USA).

For the detection and characterization of copy number variants involving the *MTM1* gene, a custom CGH array, named Motor Chip v3 and able to investigate more than 400 genes related to neuromuscular disorders with an exonic resolution [17], was used. For a refinement of the deletion detected in family VI, an ISCA v2 array was employed. CGH analyses were performed according to the manufacturer's instructions (Agilent Technologies, Santa Clara, CA, USA).

To test for skewed X-chromosome inactivation, two heterozygous microsatellites mapping on the X-chromosome (DXS8020 and DXS8015, Linkage mapping set, Applied Biosystems) were analyzed using genomic DNA from blood, muscle and buccal swab after restriction digestion with the methylation-sensitive enzyme HpaII. Analysis of the PCR products was performed on an Applied Biosystem 3130xl automated sequencer. The X inactivation pattern was defined as random at ratios 50:50, <80:20, and skewed at ratios ≥80:20 as described elsewhere [19], [20].

### 2.3 Muscle imaging, histological studies and western blot analysis

Lower limb MRI was performed in 1.5-T MR scanners as previously described [21].

Biopsies were investigated according to standard procedures [22].

For Western blot (WB) analysis skeletal muscles were homogenized in 50 mM Tris–HCl, pH 7.5, 50 mM NaCl, 10% (% v/v) glycerol, 1% (% v/v) Triton X-100 containing inhibitors of proteases (Sigma Aldrich). Samples of total protein (50 µg) were loaded and separated on 8% SDS–PAGE. Immunolabeling of the *MTM1* gene product (myotubularin) was performed by using a mouse monoclonal antibody (1:250; Abnova, Taipei City, Taiwan). The anti-GAPDH antibody (1:10,000) used as an internal control was purchased from Abcam (Cambridge, UK); peroxidase-conjugated anti-mouse IgG was used as a secondary antibody (Jackson ImmunoResearch, PA, USA). Reactive bands were detected using the Immobilon Western Chemiluminescent HRP Substrate detection kit (Millipore Corporation, Billerica, MA, USA).

## 3 Results

### 3.1 Next generation sequencing screening

To reveal the frequency of rare *MTM1* gene variants in 504 myopathic patients, we analyzed the results of a targeted resequencing study [16]: in particular, we detected 3 variants of unknown significance (VOUS – Supplementary Table S1) and 5 pathogenic variants in *MTM1*.

The c.176T>C, p.Ile59Thr was found in a female patient who presented hyperckemia and also harbored an additional, well-known, pathogenic mutation in the *DYSF* gene. The variant is listed in the ExAC database which includes 3 hemizygous individuals, reducing the possibility of a pathogenicity of this variant.

The c.1475C>T, p.Thr492Ile was detected in a healthy mother, investigated as a control in a trio analysis. Even if the variant is not present in large databases, it is predicted to be benign or neutral in 3 out of 4 programs used and the amino acid is not conserved during evolution.

The c.481G>A, p.Val161Met was detected in a 25-year-old male with a LGMD phenotype in which no further causative variants had been identified. The variant is rare (a single heterozygous individual in ExAC) but the amino acid is not conserved during evolution.

Among the 5 pathogenic variants, 4 nonsense mutations and a missense variant were identified (Table 1): two variants (c.1558C>T and c.757C>T) have already been reported [23], [24], [25], whereas the other three (c.118G>T, c.1115T>A and c.1150C>T) are novel.

### 3.2 Clinical data

All of the three male XLMTM patients identified had a similar, typical early and severe phenotype.

In particular, case II showed early neonatal hypotonia, requiring immediate respiratory support with continuous positive airway pressure and naso-jujenal feeding. Muscle biopsy revealed uniformly small myofibers and numerous fibers containing large, centrally located nuclei (75%). Some fibers contained central areas with either an excessive basophilic staining or a clearing of the cytoplasm, which correspond to aggregates of organelles on histochemical stains for reduced nicotinamide adenine dinucleotide (NADH) and cytochrome oxidase (COX). Central nuclei were found in both fiber types, and there was no evidence of grouping or other fiber type-specific changes on ATPases staining.

**Fig. 1:**
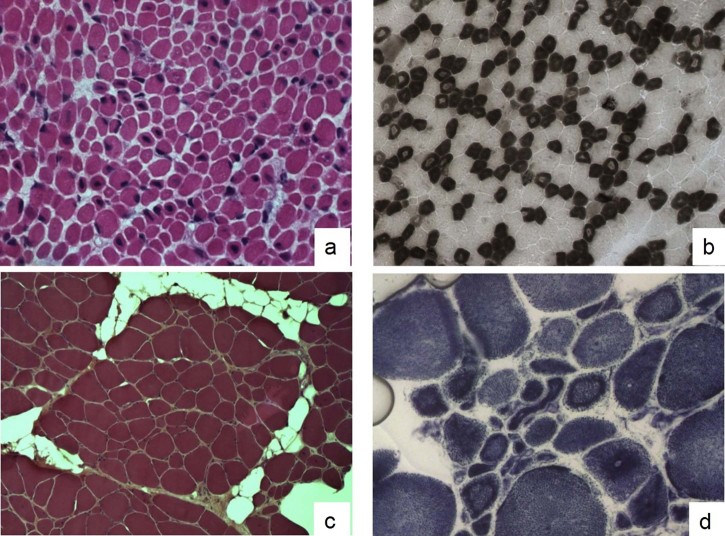
Muscle biopsies of cases IIIa and V. Muscle biopsy from patient IIIa: Hematoxylin–eosin stain (HE) (a) shows a muscle fiber size variability and many fibers (30–35%) contain a single central nucleus. ATPase 4.6 stain (b) reveals also a type 1 fiber predominance and atrophy; in particular, many fibers show a central area with a reduced myofibrillar reaction. A marked fiber size variation, several small fibers with internal nuclei and an increase of connective-adipose tissue have been observed in the HE stain (c) of a muscle biopsy from patient V. At NADH staining (d) several fibers display radial strands and internal dark ring necklace fibers.

The second patient (IIIa) was a floppy baby who presented at birth respiratory distress and feeding difficulties. His muscular biopsy revealed fiber size variability and 30–35% of the fibers containing a single central nucleus (Fig. 1a,b). We also identified two manifesting carriers in the family: the 30-year-old mother (IIIb) who has complained of mild axial and facial muscle weakness since childhood and shows elongated face and low set ears; and the 62-year-old maternal grandmother (IIIc) who has complained of mild muscle weakness since age 40 and has a waddling gait and mild facial, neck extensor muscle and mild proximal limb muscle weakness.

The last patient (case IV) was a newborn with a congenital hypotonic disorder referred to as possible myotubular myopathy. His family showed an X-linked pattern of inheritance, since three maternal uncles had reportedly early hypotonia and had died as neonates. In case IV we detected a nonsense variant in exon 9 (c.757C>T p.Arg253X) inherited from his mother.

More interestingly, we also identified two girls with a sporadic myopathy and an unremarkable family history for possible *MTM1*-related conditions or other neuromuscular disorders. Case I is a 44-year-old Italian woman, who has had, since childhood, mild leg weakness, difficulties in climbing stairs and in squatting and a characteristic tip-toe walking. Seven years ago, retraction of the Achilles' tendons was noted. Latest neuromuscular examination (July 2014) showed a facial weakness, blepharoptosis, a mild deficit of the orbicularis oculi muscles and a normal ocular motility. She also had hypotrophy of the anterior leg muscles and hypertrophy of the calves, mild retractions at the elbows and hips and a severe lumbar lordosis. Her muscle weakness was in part asymmetric, as she was significantly weaker in the muscles in her left arm and leg.

Muscle biopsy evidenced myopathic changes with several central nuclei and a normal expression of dysferlin and caveolin. Mutations in the *DMPK* gene, causing the myotonic dystrophy type 1, were excluded. Muscle MRI evidenced a severe fatty infiltration in the scapular girdle, biceps, paraspinal and abdominal muscles; a less severe infiltration of the thigh adductors and minimum and medium glutei; a more severe involvement of the anterior thigh region than the posterior; and, finally, severe fat substitution of the leg muscles (the right soleus and left medial gastrocnemius).

Patient V is a 6 year old girl from Sardinia. Her birth was uneventful but feeding difficulties appeared later. Her motor development was slightly delayed, although she could walk at 18 months. She also had mild speech delay. At the age of 3 years she was very clumsy and hypotonic.

Neurological examination at 5 years revealed a myopathic face, with a triangular mouth and a divergent strabismus in the right eye. Both proximal and distal weaknesses were present with joint hyperlaxity in her hands. Her tendon reflexes were reduced. She could rise from the floor with the Gowers maneuver and she could not run or jump. She had flat feet and mild scoliosis. Her nerve conduction velocities were normal and the EMG was consistent with myopathic damage. Her CK levels were slightly elevated (200 U/L).

**Fig. 2:**
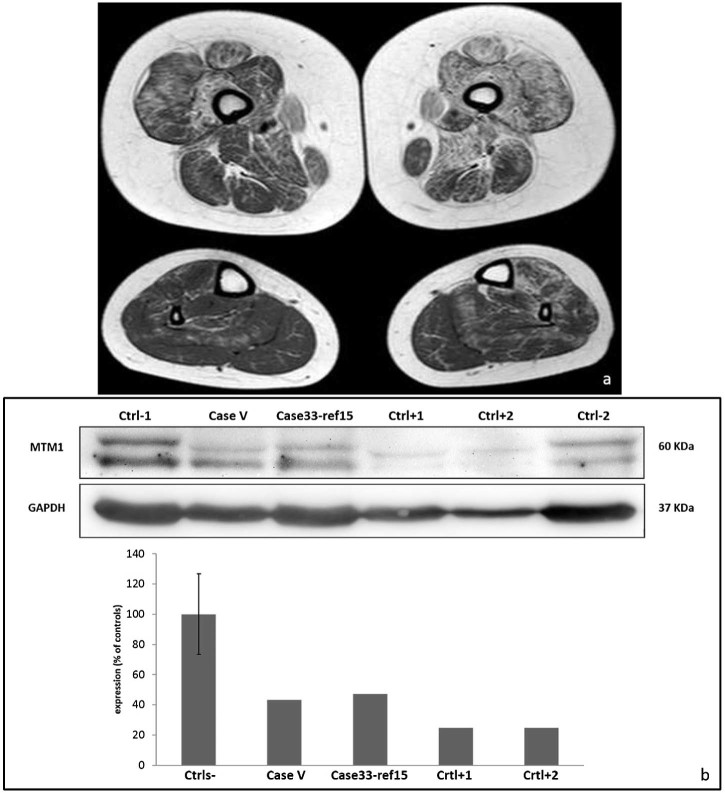
Imaging and WB of case V. In patient V (a), most muscles are affected asymmetrically (sn > dx) with only the adductor longus relatively spared. At leg level, the tibialis anterior and soleus muscles are affected on the left side. Immunolabeling and relative quantification of the *MTM1* gene product (b) show a reduced band in patient V compared to the controls.

Her muscle biopsy (Fig. 1c,d) showed a great variability in fiber diameter, an increase of connective-adipose tissue, the presence of several central nuclei and rare necklace fibers [12]. An asymmetric pattern was observed in the MRI with the tibialis anterior and soleus affected on the left side (Fig. 2a). The de novo missense variant identified in patient V results in a reduction of the protein expression (Fig. 2b), corroborating its presumable pathogenicity. Interestingly, a similar protein reduction was observed in a female patient previously reported (patient 33 in Ref. [15]) with a de novo heterozygous variant causing a misplicing.

### 3.3 Motor Chip screening for the CNV identification involving the *MTM1* gene

One hundred and five samples of patients without any significant variant detected by NGS were also analyzed by a custom CGH array, termed Motor Chip [17], but none showed a deletion or duplication involving the *MTM1* gene.

**Fig. 3:**
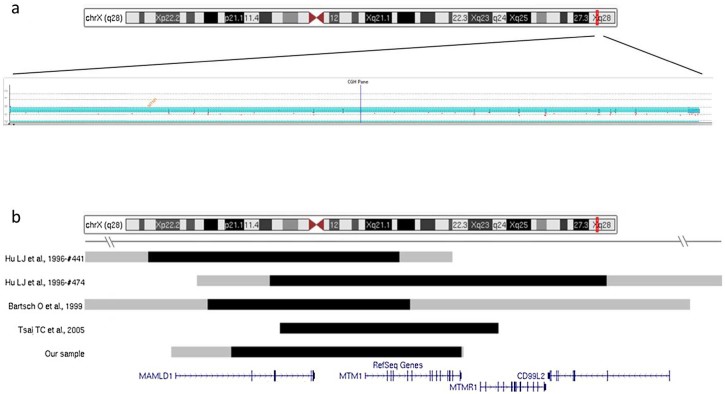
Xq28 deletions causing a myopathy with an abnormal genital development. The Motor Chip results evidence the presence of a large deletion in the mother of patient VI (a). The graphical representation (b) shows the extent of the deletion region identified in patient VI, described in this study, and in the four patients previously reported. Black lines indicate the minimum deletion area; gray, the maximum deletion region.

We detected a deletion of the chromosomal region comprising the *MTM1* gene in DNA from a healthy woman who had sought prenatal counseling to assess the risk of recurrence for a complex phenotype that had been observed in her first son. This child had shown, after delivery, a very severe phenotype, including general hypotonia, an ogival palate, hypospadias, scrotal hypoplasia, a single umbilical artery, camptodactyly and clubfoot. At counseling, a written report evidencing an entire deletion of the *MTM1* gene detected by MLPA was provided. We identified a heterozygous deletion at Xq28 encompassing *MTM1* and additional genomic regions in the DNA from the mother (minimum deletion region: chrX: 149591931–149841591; maximum deletion region: chrX: 149526823–149844072) (Fig. 3a). The deletion included the MAMLD1 (CXORF6 or F18) gene, already described as part of a contiguous gene syndrome in two unrelated children [26], [27] who presented at birth with hypotonia and abnormal genitalia. The association of this syndromic muscular phenotype has later been corroborated in two further independent cases [28], [29]. Thus our description represents the fifth large deletion on Xq28 (Fig. 3b).

## 4 Discussion

*MTM1* mutations are associated with the X-linked myotubular myopathy-1, a congenital myopathy, with typical histological findings of central nuclei, hypotrophy and a predominance of type I fibers.

A neonatal onset of the disease, as further corroborated by our investigations, in a male newborn should be sufficient to lead clinicians toward a hypothesis of an X-linked myotubular myopathy and to request a direct *MTM1* analysis. Similarly, the presence of a congenital myopathy with anomalies of the genitalia should always address the testing toward a CNV analysis.

Our large NGS screening had been preceded by gene-specific tests in agreement with the clinical and histological findings. It is reasonable to think that, for most male patients with a very early severe onset, the *MTM1* gene will have already been analyzed: only patients negative at this first tier screening will have been referred to our center, justifying the low number of positive patients identified. On the other hand, family III is quite particular: male patient IIIa, showing a severe congenital myopathy and a premature death, had an affected mother (IIIb) and an affected maternal grandmother (IIIc). His family history may have initially suggested an autosomal dominant transmission with anticipation in alternative to an X-linked condition and precluded direct gene testing.

The identification of two (I, V) female patients with *MTM1* mutations in the absence of affected males represents an important result highlighting the need for a broad multigene study. Even if several different symptomatic carriers of *MTM1* variants have been described in the literature (Table 2), their detection usually derived from the diagnosis of an affected male, as in the case of IIIb and IIIc.

Moreover, a skewed X-inactivation has been considered as the cause of the phenotype in female carriers. However, a re-evaluation of the previously published data seems not to support this hypothesis: in the 5 year old girl with hypotonia and general weakness, described by Schara et al. [34], a unilateral X-inactivation was detected in the muscles but not in the blood; eight females showed a random expression [32], [33], [35], [36], [37], [38]; and in only 5 cases was a skewed expression confirmed in the blood [19], [30], [31], [35], [37]. In addition, in our case V, a skewed inactivation was not observed (ratio < 60:40). We also reevaluated the X-inactivation in a female patient, already published in a previous paper (patient 33 in Ref. [15]), detecting a random inactivation (ratio < 60:40). It is anyway reasonable to assume that the skewed X-inactivation may be at least one of the reasons leading to the presence of manifesting carriers but other epigenetic factors may be involved in the X inactivation of the normal allele.

However, the penetrance of XLMTM in females is reported to be lower than that of other muscular disorders, such as the Duchenne and Becker muscular dystrophies in which 22% of female carriers are symptomatic or the Emery-Dreifuss muscular dystrophy where 18% of female carriers have ECG abnormalities [39].

On the other hand, our study evidences that the frequency of symptomatic female carriers may be underestimated. All of the female patients with MTM1 mutations included in the paper showed histological features related to a centronuclear myopathy, confirming previous published data [15]. Nowadays, however, NGS screenings represent a straightforward approach that is revolutionizing the genetic diagnosis also in the field of neuromuscular disorders [16], [40]. Therefore, large NGS panels may be considered useful tools to identify sporadic female XLMTM and the *MTM1* gene should always be included in any NGS test performed for the diagnosis of neuromuscular disorders.

Even if our Motor Chip analyses did not evidence any variant in the gene, the possible presence of a heterozygous deletion or duplication, accounting for about 7% of *MTM1* defects [11] should be always considered because of the NGS limitation in detecting heterozygous CNV. Moreover, considering the low penetrance of mutated alleles in females, any variant identified in the *MTM1* gene in the NGS analyses represents an incidental finding which should be reported to the patient.

## Figures and Tables

**Table 1:** List of patients.

ID	Sex	Age of onset	Age of death	Severity	Transmission	Histological features	CK levels	Mutation	References
I	Female	Childhood	–	Intermediate	Sporadic	Several central nuclei	Normal	c.118G>T, p.(Gly40X)	Novel
II	Male	Birth	3 mo.	Severe	Sporadic	Small myofibers and a numerous fibers containing large, centrally located nuclei (75%)	Normal	c.1558C>T, p.(Arg520X)	Described (Ref. [23])
IIIa	Male	Birth	3 wks	Severe	Dominant/X-linked	More than 50% of fibers with myotubular appearance, marked predominance of type I fibers	Normal	c.1150C>T, p.(Gln384X)	Novel
IIIb	Female	Childhood	–	Mild	Few fibers with central nuclei marked predominance of type I fibers	Normal
IIIc	Female	40 yrs	–	Intermediate	Few fibers with central nuclei marked predominance of type I fibers , small type grouping	Normal
IV	Male	Birth	2 mo.	Severe	X-linked	Several central nuclei	n.a.	c.757C>T, p.(Arg253X)	Described (Refs. [24], [25])
V	Female	Infancy	–	Intermediate	Sporadic	Variability in fiber diameter with several central nuclei and necklace fibers	slightly elevated	c.1115T>A, p.(Leu372His)	Novel
VI	Male	Birth	n.a.	Severe	X-linked	n.a.	n.a.	Interstitial deletion at Xq28	Novel

n.a. = not available; mo. = months; wks = weeks; yrs = years.

**Table 2:** MTM1 female symptomatic carriers.

Patient	Age of onset	Mutation	X-chromosome inactivation	References
1	Infancy	Del Xq27-q28	Skewed^b^	Dahl et al. [30]
2	Childhood	c.1261-10A>G, p.(Ser420_Arg421insPheIleGln)	Skewed^b^	Tanner et al. [31]
3	Childhood	c.1132G>A, p.(Gly378Arg)	Random^b^	Hammans et al. [32]
4	Childhood	c.670C>T, p.(Arg224X)	Random^b,m^	Sutton et al. [33]
5	Infancy	c.605delT , p.(Leu202TrpfsX48)	Random^b^ – Skewed^m^	Schara et al. [34]
6	Infancy	c.1261C>T, p.(Arg421X)	Skewed^b^	Jungbluth et al. [19]
7	Childhood	c.757C>T, p.(Arg253X)	Random^b,m^	Grogan et al. [35]
8	Childhood	c.757C>T, p.(Arg253X)	Not informative	Grogan et al. [35]
9	Childhood	c.757C>T, p.(Arg253X)	Random^b^	Grogan et al. [35]
10	Childhood	c.1354-1G>A, p.(Phe452fs)	Skewed^b^	Grogan et al. [35]
11	Childhood	c.1493T>A, p.(Leu498X)	Random^b,m^	Penisson-Besnier et al. [36]
12	Childhood	c.1420C>T, p.(Arg474X)	Random^b^	Drouet et al. [37]
13	Infancy	c.1420C>T, p.(Arg474X)	Random^b^	Drouet et al. [37]
14	Adult	c.1420C>T, p.(Arg474X)	Skewed^b^	Drouet et al. [37]
15	Childhood	c.205_206delinsAACT, p.(Arg69AsnfsX5)	n.a.	Bevilacqua et al. [12]
16	Childhood	c.1262G>A, p.(Arg421Gln)	n.a.	Bevilacqua et al. [12]
17	Childhood	c.1234A>G, p.(Ile412_Ser420del)	n.a.	Bevilacqua et al. [12]
18	Childhood	c.867+1G>T, p.(Val227_Lys289del)	Random^m^	Hedberg et al. [38]
19	Childhood	c.867+1G>T, p.[Val227_Lys289del, Val227_Lys351del]	Not informative	Hedberg et al. [38]
20	Infancy	c.417A>G, p.(=), r.(spl?)	Random^b,m,sw^	Fattori et al. [15]
21	Childhood	c.118G>T, p.(Gly40X)	n.a.	pt I (this study)
22	Childhood	c.1150C>T, p.(Gln384X)	n.a.	pt IIIb (this study)
23	Adult	c.1150C>T, p.(Gln384X)	n.a.	pt IIIc (this study)
24	Infancy	c.1115T>A, p.(Leu372His)	Random^b,m,sw^	pt V (this study)

b = blood; m = muscle; sw = buccal swab; n.a. = not available.

## Appendix Supplementary material

Table S1 Variants of uncertain significance (VOUS).
